# Different Pattern of Immunoglobulin Gene Usage by HIV-1 Compared to Non-HIV-1 Antibodies Derived from the Same Infected Subject

**DOI:** 10.1371/journal.pone.0039534

**Published:** 2012-06-25

**Authors:** Liuzhe Li, Xiao-Hong Wang, Sagarika Banerjee, Barbara Volsky, Constance Williams, Diana Virland, Arthur Nadas, Michael S. Seaman, Xuemin Chen, Paul Spearman, Susan Zolla-Pazner, Miroslaw K. Gorny

**Affiliations:** 1 Department of Pathology, New York University School of Medicine, New York, New York, United States of America; 2 Veterans Affairs New York Harbor Healthcare System, New York, New York, United States of America; 3 Institute of Environmental Medicine, New York University School of Medicine, New York, New York, United States of America; 4 Beth Israel Deaconess Medical Center, Harvard Medical School, Boston, Massachusetts, United States of America; 5 Department of Pediatrics, Emory University, Atlanta, Georgia, United States of America; National Institute of Infectious Diseases, Japan

## Abstract

A biased usage of immunoglobulin (Ig) genes is observed in human anti-HIV-1 monoclonal antibodies (mAbs) resulting probably from compensation to reduced usage of the VH3 family genes, while the other alternative suggests that this bias usage is due to antigen requirements. If the antigen structure is responsible for the preferential usage of particular Ig genes, it may have certain implications for HIV vaccine development by the targeting of particular Ig gene-encoded B cell receptors to induce neutralizing anti-HIV-1 antibodies. To address this issue, we have produced HIV-1 specific and non-HIV-1 mAbs from an infected individual and analyzed the Ig gene usage. Green-fluorescence labeled virus-like particles (VLP) expressing HIV-1 envelope (Env) proteins of JRFL and BaL and control VLPs (without Env) were used to select single B cells for the production of 68 recombinant mAbs. Ten of these mAbs were HIV-1 Env specific with neutralizing activity against V3 and the CD4 binding site, as well as non-neutralizing mAbs to gp41. The remaining 58 mAbs were non-HIV-1 Env mAbs with undefined specificities. Analysis revealed that biased usage of Ig genes was restricted only to anti-HIV-1 but not to non-HIV-1 mAbs. The VH1 family genes were dominantly used, followed by VH3, VH4, and VH5 among anti-HIV-1 mAbs, while non-HIV-1 specific mAbs preferentially used VH3 family genes, followed by VH4, VH1 and VH5 families in a pattern identical to Abs derived from healthy individuals. This observation suggests that the biased usage of Ig genes by anti-HIV-1 mAbs is driven by structural requirements of the virus antigens rather than by compensation to any depletion of VH3 B cells due to autoreactive mechanisms, according to the gp120 superantigen hypothesis.

## Introduction

Neutralizing antibodies (Abs) are critical elements in vaccine development as they form the first line of defense against pathogens and are associated with protection against virus infection [1]. The role of Abs in preventing infection with HIV [2], [3], [4], simian immunodeficiency virus (SIV) [5], and simian/human immunodeficiency virus (SHIV) [6], [7] has been firmly established by several passive immunization experiments in various animal models. However, generating protective Ab responses has proven to be an enormous challenge because the available vaccine immunogens elicit Abs that neutralize only a minority of HIV-1 isolates [8].

Searching for the cause of the relatively ineffective neutralizing activity of anti-HIV-1 Abs, attention was turned towards the immunoglobulin (Ig) genes coding for these Abs. Immunogenetics studies revealed biased Ig gene usage by anti-HIV-1 mAbs, including neutralizing mAbs [9], [10]. Ig variable genes coding for heavy chains are used by human anti-HIV-1 mAbs with different frequencies compared to Abs from healthy individuals. The canonical VH3 family genes are used significantly less frequently by anti-HIV-1 mAbs, while VH1 family genes are preferentially used by mAbs against CD4i, gp41 and some other anti-HIV-1 envelope (Env) mAbs [10], [11], [12], [13], [14]. Furthermore, we have shown that anti-V3 mAbs preferentially use the VH5-51 gene segment [9], [15]. This suggests that biased usage of Ig genes may depend on antigen requirements and that only certain Ig gene-encoded Abs fit well and with high initial affinity to Env antigens. If this hypothesis is correct, then targeting such Ig genes may trigger Abs with enhanced affinity maturation to the HIV-1 epitopes.

It was also hypothesized that the selective depletion of the canonical VH3 family genes due to autoreactivity towards B cells may result in the preferential usage of other VH families for anti-HIV-1 Abs by way of compensation. It has been shown that gp120 behaves as a superantigen which binds to B cell receptors encoded by VH3 genes and such cells can be recognized as HIV-1 infected and eliminated by the immune effector cells [16].

To test these hypotheses, we generated mAbs from single B cells derived from an HIV-1 infected individual using for selection green-fluorescent protein-labeled (GFP) virus-like particles (VLPs) expressing Env antigens. The VLPs have been previously utilized in the production of human mAbs against rotavirus [17]. A similar method using VLPs expressing HIV-1_BaL_ Env proteins has also been used to produce a human anti-CD4 induced antigen (CD4i) mAb [18].

In total, 68 mAbs were produced, including 10 HIV-1 Env specific mAbs against V3, CD4-binding site (CD4bs) and gp41, as well as 58 non-HIV-1 mAbs selected by Env- and non-Env expressing GFP-VLPs, respectively. Analysis of Ig genes used by HIV-1 specific mAbs compared to non-HIV-1 mAbs with undefined specificities revealed that the biased usage of Ig genes is restricted to anti-HIV-1 Env mAbs only, whereas non-HIV-1 mAbs present Ig gene utilization patterns identical to that of healthy individuals. This suggests that the biased usage of Ig genes by anti-HIV-1 mAbs results from structural requirements of the virus antigens, not from other mechanisms.

## Results

### Single Memory B Cell Sorting

**Figure 1:**
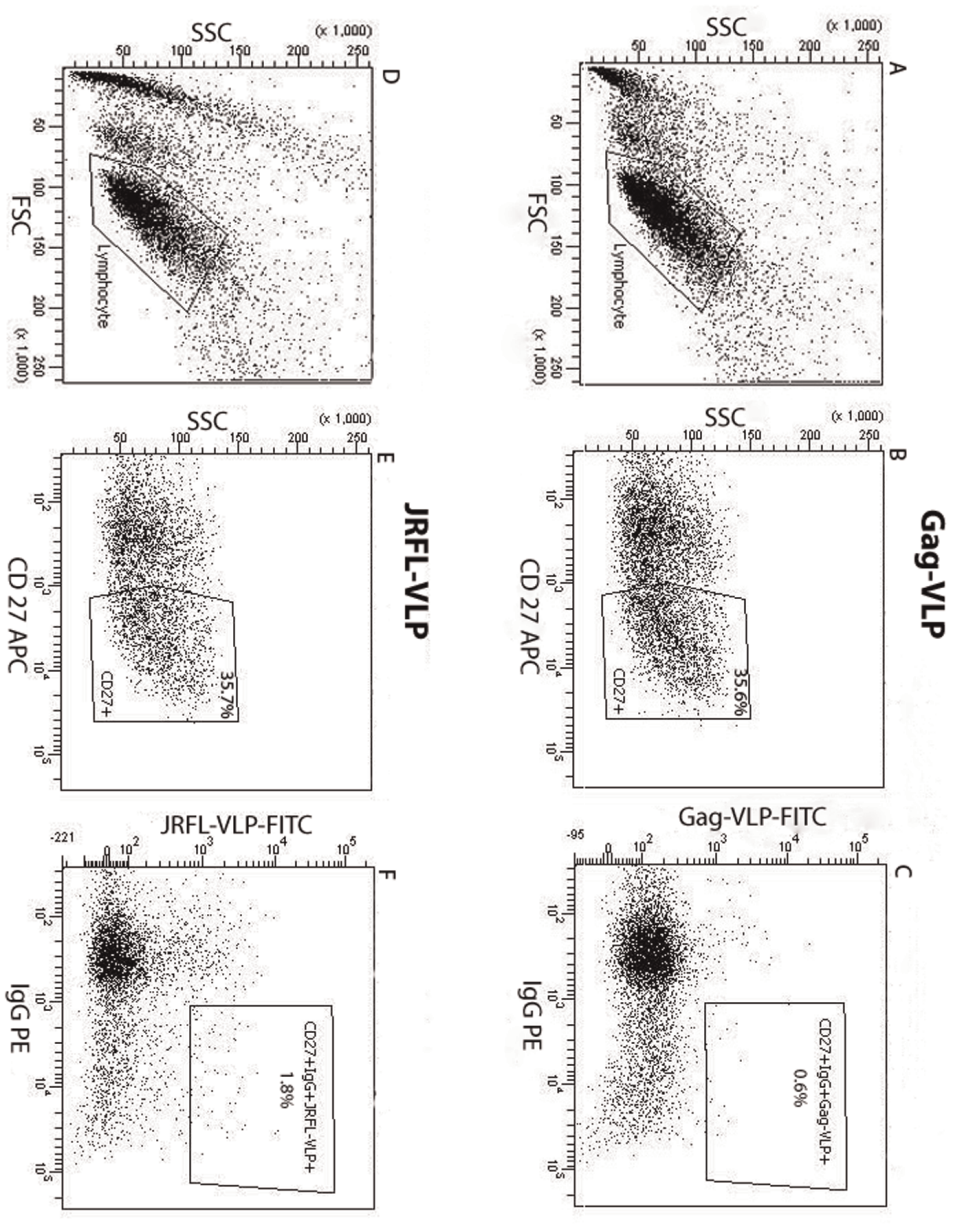
Cell sorting of B cells stained with VLPs, anti-CD27, and anti-IgG . The top panel (Gag-VLP) and bottom panel (JRFL-VLP) indicate the gating of non-HIV-1 and anti-HIV-1 Env Abs expressing B cells, respectively. (1A and 1D) FSC and SSC show forward scatter and side scatter, measures of cell size and granularity. The selected area shows the gated single live cells from CD19 magnetic beads enriched B cells. (1B and 1E) The dot plots show the percentages of CD27^+^ memory B cells. Numbers indicate the percentage of gated cells stained with anti-CD27-APC. The percentages of CD27^+^ memory B cells are similar prior to VLPs^+^ selection in both non-HIV-1 and anti-HIV Env Abs expressing cells. (1C and 1F) The dot plots show the gating of IgG^+^ and Gag-VLP^+^ cells (1C) or IgG^+^ and JRFL-VLP^+^ cells (1F) on the CD27^+^ memory B cells. The selected area shows percent of total B cells stained for Gag-VLP (1C) and JRFL-VLP (1F).

VLPs expressing the Env proteins of JRFL and Bal, as well as Gag-VLP (without Env) negative controls, were used to singly sort out the Env-specific and non-HIV-1 Env specific IgG^+^ memory B cells, respectively, for production of human mAbs. PBMCs were purified from the blood sample of one NYU IRB-approved HIV-1 infected volunteer. The B cells were enriched by using CD19 magnetic beads, and incubated with VLPs tagged with green fluorescent protein (GFP) followed by staining with PE-anti-IgG and APC-anti-CD27. The B cells were stained separately for Gag-VLPs (without Env proteins) (Fig. 1A, B and C) and for VLPs expressing JRFL Env (Fig. 1D, E and F) and were then singly sorted into 96-well plates. The Gag-VLPs stained 0.6% (Fig. 1C) of total cells as background, while JRFL-VLPs stained 1.8% (Fig. 1F) and BaL-VLPs stained 1.2% (data not shown) of total cells.

### Engineered mAbs from Single Memory B Cells

Amplified PCR products of the heavy and light chain variable regions from single cells selected by JRFL-VLPs, BaL-VLPs and control Gag-VLPs were sequenced and compared with germline sequences in the IMGT database. Functional heavy and light chain variable gene sequences were separately cloned into expression vectors containing the constant regions of γ1, κ and λ [19]. Heavy and light chain constructs from the same single B cell were then cotransfected into 293T cells to produce mAbs.

In total, 68 culture supernatants from day three transfected 293T cells contained IgG, as determined by a quantitative ELISA, in the range of 0.5 µg/ml to 50 µg/ml with the majority of samples between 10–20 µg/ml (data not shown). The supernatants were adjusted to 10 µg/ml, excepting those which were <10 µg/ml, and screened by ELISA against recombinant gp120_MN_ and recombinant gp41_MN_. Ten mAbs selected by JRFL-VLPs were reactive with HIV-1 Env proteins while 58 recombinant mAbs did not display any reactivity to viral Env proteins, and their specificity was not determined (Table 1 and 2). Using BaL-VLPs no HIV-1 specific mAbs were selected possibly due to low density of cleaved gp120 on the surface of VLPs which was observed by Western blot analysis [18]. These 58 non-HIV-1 mAbs included 24 selected with JRFL-VLPs, 19 with BaL-VLPs and 15 mAbs with Gag-VLPs which were used as negative controls (Table 2).

**Table 1:** Anti-HIV-1 envelope mAbs produced from single IgG^+^ memory B cells selected using JRFL expressing virus-like particles.

#	mAb	Site	ELISAgp120_MN_O.D.^1^	ELISAgp41_MN_O.D.^1^	JRFL/293T%^2^	SF162/293T%^2^	(-)/293T%^2^	ELISAcardiolipin;O.D.^1^
1	3b5	V3	**3.6**	0.1	**5.3**	**44.6**	0.8	0.1
2	3b38	V3	**3.7**	0.1	**8.4**	**46.9**	2.0	0.2
3	3b96	V3	**3.7**	0.1	**25.2**	**49.9**	**6.6**	0.2
4	3c50	CD4bs	**3.5**	0.1	**3.6**	**47.8**	1.6	0.2
5	3c25	CD4bs	**2.3**	0.1	**3.4**	**35.3**	1.7	**0.3**
6	3c81	CD4bs	**2.1**	0.1	**4.4**	**46.6**	2.1	0.1
7	3c53	gp41	0.1	**3.7**	**24.2**	**27.1**	**12.1**	**2.2**
8	3b95	gp41	0.1	**1.9**	**7.1**	**16.8**	**3.0**	**2.0**
9	3c16	gp41	0.1	**3.0**	1.2	**14.2**	1.2	0.2
10	3c91	gp41	0.1	**1.0**	1.9	2.5	2.0	**0.3**
C (+)	447	V3	**3.5**	0.1	**35.7**	**56.1**	**11.3**	0.1
C (+)	654	CD4bs	**1.9**	0.1	nt	nt	1.6	nt
C (+)	167	gp41	0.1	**3.3**	nt	nt	2.2	**2.3**
C (+)	serum	–	nt	nt	nt	nt	nt	**1.2**
C (−)	1418	B19	0.1	0.1	1.2	1.3	1.2	0.1
C (−)	serum	–	nt	nt	nt	nt	nt	0.1

1 A standard ELISA was used to determine the binding activity of mAbs to antigens. Monoclonal Abs were tested at a concentration of 10 µg/ml against gp120 and gp41 coated onto ELISA plate at 1 µg/ml; cardiolipin at a concentration of 45 µg/ml in ethanol was coated by evaporation at 4°C overnight. The numbers are O.D. values; bold numbers indicate specific reactivity based on value above cutoff which was defined as the mean binding of mAb 1418 (specific to parvovirus B19) +3 standard deviations. ^2^Flow cytometry was used to measure the mAb binding to JRFL, SF162 Env-transfected 293T cells and native 293T cells as control. The bold numbers indicate percent of cells specifically reactive with mAbs based on values above cutoff determined with irrelevant mAb 1418 as described above. C(+) – positive control; C(-) – negative control; nt – not tested.

**Table 2:** Biased usage of VH family genes by anti-HIV-1 mAbs produced from one infected individual.

VH family^1^	JRFL-VLPsanti-HIV-1	JRFL-VLPsnon-HIV-1 env	BaL-VLPsnon-HIV-1 env	(-)Gag-VLPsnon-HIV-1
1	**4 (40%)**	5 (21%)	–	4 (27%)
3	3 (30%)	**12 (50%)**	**12 (63%)**	**7 (47%)**
4	2 (20%)	6 (25%)	6 (32%)	3 (20%)
5	1 (10%)	1 (4%)	1 (5%)	1 (6%)
Total No.	10	24	19	15
of mAbs		68		

1 VH family 2, 6 and 7 were not detected; predominantly used VH family genes by mAbs are in bold type.

### Anti-HIV-1 Env mAbs

Ten HIV-1 Env specific mAbs were further tested in a quality control assay (QC) against seven HIV-1 antigens: recombinant gp120_MN_, V3_B_-cholera toxin B (CTB) fusion protein, V3_C_-CTB, C5 peptide (aa 495–516), recombinant gp41_MN_, recombinant p24 and bovine serum albumin (BSA) (data not shown). Three mAbs, 3b5, 3b38 and 3b96, were specific to the V3 region and bound to both gp120 (Table 1) and V3-CTBs. Another three mAbs, 3c25, 3c50, and 3c81, were reactive only with gp120 (Table 1) in the QC assay. In an additional assay they were reactive with wild type gp120_HXB2_ but not (3c25 and 3c81) or partially (3c50) with the D368R + N448Q mutant of gp120_HXB2_, confirming their specificity to CD4bs (data not shown). This was also consistent with the significant reduction of the binding of 3c25 and 3c81, but not 3c50, to gp120 by 2 µg/mL sCD4 (Fig. S1). It suggested that 3c25 and 3c81 mAbs recognize the epitope in the CD4bs while the epitope of 3c50 overlaps with CD4bs. Both, anti-V3 and anti-CD4bs mAbs were reactive with 293T cells transfected with Env proteins of JRFL and SF162.

The remaining four mAbs, 3c16, 3c53, 3c91 and 3b95, were specific to gp41 based on binding to recombinant gp41_MN_ only (Table 1). The epitope of all four mAbs has a conformational character, as they were only reactive with the native recombinant gp41, but not with denatured gp41 which was treated with dithiothreitol and boiled at 95°C (data not shown). Binding of gp41 mAbs to 293T cells transfected with the Env JRFL and SF162 showed their mixed reactivity: in particular, mAbs 3c16 and 3c91 were weakly reactive or nonreactive, possibly due to limited exposure of the epitope in the context of the trimer (Table 1).

The epitope location in gp41 was tested in a competition assay using biotinylated mAbs 50–69 (specific to cluster I a.a. 579–604) and 167 (specific to cluster II a.a.644–663) [20]. One mAb, 3c16, inhibited binding of both biotinylated 167 and 50–69 to gp41, suggesting that it might bind to one of the epitope clusters, either I or II, which are very close to each other due to the lysine finger interaction between heptad repeat 1 and 2 of gp41. A similar phenomenon was recently reported with two recombinant anti-gp41 mAbs which were found to compete with anti-cluster I and IV biotinylated mAbs [10]. The remaining three anti-gp41 mAbs, 3c53, 3b95, and 3c91, displayed no inhibitory activity to the two biotinylated mAbs, suggesting that their antigenic determinants are located outside of the cluster I and II epitopes in gp41 (data not shown).

### Autoreactivity of Anti-HIV-1 mAbs

Testing of mAb binding to Env-transfected 293T cells includes some possibility of false positive reactivity dependant on the binding to cell components, but not to Env proteins. It was shown that 3 of 10 mAbs, 3b96, 3c53, and 3b95, had some binding activity toward untransfected 293T cells, in the range of 3% to 12.1%. However this level of binding activity is 2 to 7.5 fold lower than binding to Env-transfected cells (Table 1). One of the control mAbs, 447-52D (anti-V3), also exhibited some non-specific reactivity with untransfected 293T cells, with 3.1 and 4.9 fold lower binding than to JRFL- and SF162-Env transfected cells, respectively (Table 1).

In addition, all mAbs were tested against cardiolipin. Two gp41 mAbs, 3c53 and 3b95, displayed reactivity to both cardiolipin and 293T cells, which suggests that they may bind to phospholipids present in the cell membrane. Two other mAbs, 3c25 and 3c91, exhibited borderline binding activity with cardiolipin (Table 1).

### Non-HIV-1 Env mAbs

Fifty-eight mAbs selected with JRFL-VLPs, BaL-VLPs and control Gag-VLPs were non-binding to gp120MN, gp41MN and to JRFL Env transfected 293T and they were named the non-HIV-1 Env mAbs (data not shown). Three mAbs, 3c11, 3c13 and 3d89, were exceptional as they did not bind to gp120MN and gp41MN but reacted with both JRFL Env transfected and native 293T cells exhibiting some autoreactive characteristics (data not shown).

### Neutralizing Activity of gp120-specific mAbs

All 10 mAbs specific to the V3, CD4bs and gp41 epitopes were screened for neutralizing activity in the TZM-bl cell assay against SF162 and 6535 pseudoviruses. Six mAbs against V3 and CD4bs (3b38, 3b96, 3b5, 3c50, 3c81 and 3c25) neutralized both viruses (data not shown) and were further tested against a panel of 41 pseudoviruses, with the exception of mAb 3b96 which was tested with 23 pseudoviruses (Table 3). Generally, one or more mAbs neutralized the majority of tier 1 viruses (10 of 15) while tier 2 viruses were more resistant, probably due to virus-mediated masking mechanisms, as only four of 26 were neutralized (Table 3). All three anti-V3 mAbs and one anti-CD4bs mAb, 3c50, cross-neutralized a comparable number of pseudoviruses, between six and nine of 41 tested, while the two remaining anti-CD4bs mAbs, 3c25 and 3c81, neutralized only three and two viruses, respectively (Table 3). These two anti-CD4bs mAbs displayed possibly lower affinity binding as they reacted with gp120 relatively weakly (Table 1, Fig. S1).

**Table 3:** Neutralization of pseudoviruses by anti-V3 and anti-CD4bs mAbs.

				V3			CD4b		B19
Virus	Tier	Clade	3b38	3b96	3b5	3c50	3c81	3c25	1418
Bx08.16	1B	B	**<0.4**	**<0.4**	**0.8**	**2.1**	**5.2**	**10.6**	>50
SF162.LS	1A	B	**<0.4**	**<0.4**	**<0.4**	**0.5**	**1.2**	>50	>50
BaL.26	1B	B	**0.6**	**<0.4**	**1.2**	**2.3**	>50	**6.3**	>50
SS1196.1	1B	B	**1.6**	**0.5**	**3.3**	**11**	>50	>50	>50
MW965.26	1A	C	**0.4**	**15**	**3.1**	>50	>50	>50	>50
271-11	1B	AG	>50	**0.6**	**25.9**	>50	>50	>50	>50
HXB2.DG	1B	B	>50	nt	>50	**<0.4**	**0.6**	nt	>50
6535.3	1B	B	>50	**2.1**	>50	**3.4**	>50	>50	>50
DJ263.8	1B	A	**0.7**	>50	>50	>50	>50	>50	>50
BZ167.12	1B	B	**1.3**	>50	>50	>50	>50	>50	>50
HO31.7	2	B	**14.6**	nt	>50	>50	>50	>50	>50
JRFL.JB	2	B	**49.9**	nt	>50	>50	>50	nt	>50
WITO4160.33	2	B	>50	**38.3**	>50	>50	>50	>50	>50
QH0692.42	2	B	>50	nt	**43.7**	>50	>50	>50	>50
25710-2.43	1B	C	>50	>50	>50	>50	>50	>50	>50
ZM109F.PB4	1B	C	>50	nt	>50	>50	>50	>50	>50
ZM197M.PB7	1B	C	>50	>50	>50	>50	>50	>50	>50
242-14	1B	AG	>50	>50	>50	>50	>50	>50	>50
HO29.12	1B	B	>50	nt	>50	>50	>50	>50	>50
HO30.7	2	B	>50	nt	>50	>50	>50	>50	>50
AC10.0.29	2	B	>50	>50	>50	>50	>50	>50	>50
CAAN5342.A2	2	B	>50	>50	>50	>50	>50	>50	>50
REJO4541.67	2	B	>50	>50	>50	>50	>50	>50	>50
RHPA4259.7	2	B	>50	>50	>50	>50	>50	>50	>50
SC422661.8	2	B	>50	>50	>50	>50	>50	>50	>50
TRO.11	2	B	>50	>50	>50	>50	>50	>50	>50
THRO4156.18	2	B	>50	>50	>50	>50	>50	>50	>50
CAP45.2.00.G3	2	C	>50	nt	>50	>50	>50	>50	>50
CAP210.2.00.E8	2	C	>50	nt	>50	>50	>50	>50	>50
Du156.12	2	C	>50	nt	>50	>50	>50	>50	>50
Du172.17	2	C	>50	nt	>50	>50	>50	>50	>50
Du422.1	2	C	>50	nt	>50	>50	>50	>50	>50
ZM53M.PB12	2	C	>50	nt	>50	>50	>50	>50	>50
ZM135M.PL10	2	C	>50	nt	>50	>50	>50	>50	>50
ZM214M.PL15	2	C	>50	nt	>50	>50	>50	>50	>50
ZM233M.PB6	2	C	>50	>50	>50	>50	>50	>50	>50
ZM249M.PL1	2	C	>50	nt	>50	>50	>50	>50	>50
HO35.18	3	B	>50	nt	>50	>50	>50	>50	>50
HO61.14	3	B	>50	nt	>50	>50	>50	nt	>50
PVO.4	3	B	>50	>50	>50	>50	>50	>50	>50
TRJO4551.58	3	B	>50	>50	>50	>50	>50	>50	>50

1 Neutralization of pseudoviruses was performed using the TZM-bl cell assay. All mAbs were titrated by 2-fold serial dilutions from maximum concentration of 50 µg/ml. The number above represent the concentration of mAb needed for 50% neutralization (IC50); the bold number indicates mAb with neutralizing activity above 50%. Monoclonal antibody 1418 (against parvovirus B19) was used as negative control. nt – not tested.

### Biased Usage of Immunoglobulin Genes by Anti-HIV-1 mAbs Compared to Other mAbs

**Figure 2:**
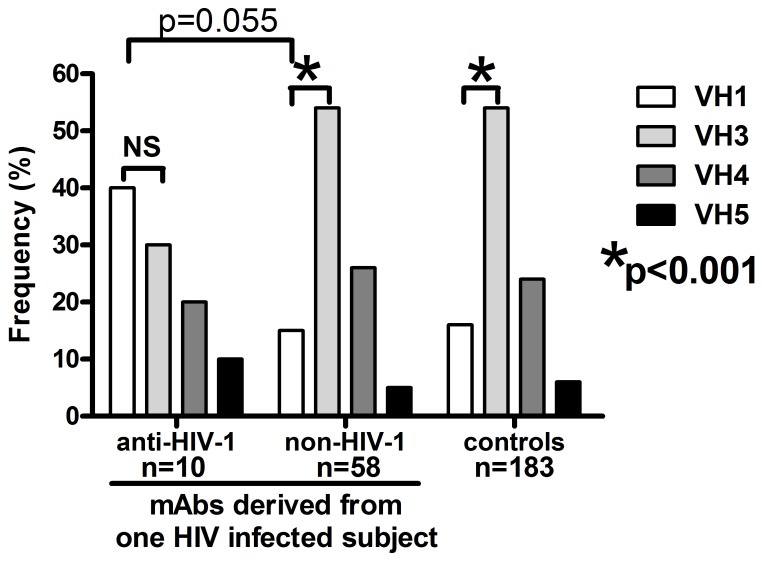
The usage of VH family genes by human anti-HIV-1 and non-HIV-1 mAbs. These mAbs were produced from single B cells derived from one HIV infected individual and are compared to antibodies with undefined specificities produced from single B cells of four healthy control subjects [21]. The preferential usage of the VH3 versus VH1 family genes by non-HIV-1 mAbs compared controls and significantly increased usage of VH1 family genes of anti-HIV-1 versus non-HIV-1 mAbs was determined by the Chi-Squared test. NS – not significant.

Production of 68 mAbs from one infected individual, with different specificities to HIV-1 and non-HIV-1 Env antigens, allowed for the comparison of the Ig variable genes usage by these two different panels of mAbs. Anti-HIV-1 Env mAbs selected by the JRFL-VLPs predominantly used VH1 family genes (four of ten mAbs used VH1 gene, 40%) over VH3 family genes (three of 10 mAbs used VH3 gene, 30%), while VH4 and VH5 family genes were used by 20% and 10% of mAbs, respectively (Fig. 2, Table 4, Table 2).

**Table 4:** Anti-HIV-1 envelope mAbs produced from single IgG^+^ memory B cells selected using JRFL expressing virus-like particles.

#	mAb	Site	IGHV	CDR H3	IGLV	CDR L3
1	3b5	V3	3–30	AAHYDSYGLNV	L1–51	GSWDGGPNLGWV
2	3b38	V3	5–51	ARQGDRSGYDF	L6–57	QSYDDTSYV
3	3b96	V3	1–69	VRDGDVGDH	L2–14	NSYTSSKSVI
4	3c81	CD4bs	1–18	ARRRAGYGWGSDYSDGFYFDY	K1–5	QQYNSYPT
5	3c50	CD4bs	1–69	ARERLHARGPLGTRYYGLDV	K3–15	QHYNSWPRT
6	3c25	CD4bs	4–59	TRDSPKRYSYDRRHYYYFGLDV	L2–14	NSHTSSGTLV
7	3c16	gp41	1–69	ARARRDGLLFTFDN	K1–16	QQYNSYPPT
8	3c53	gp41	3–30	AKDRSSSNWYEYYYGMDV	L1–44	AAWDDILNGWV
9	3c91	gp41	3–23	AKDARPKTRYYDILTGYYSPEGDYYAMDV	L3–1	QAWDSSTACV
10	3b95	gp41	4–31	ARGRPHNRYSTRAYFDY	K3–20	QQYGSSPPIT

The sequences have been submitted to GenBank (accession numbers: JQ301900– JQ301919).

The non-HIV-1 mAbs with undefined specificity selected by JRFL-VLPs (N = 24), Bal-VLPs (N = 19) and Gag-VLPs (N = 15) (Table 2) predominantly used the VH3 family genes (mean 54%) followed by VH4 genes (25%), while VH1 and VH5 family genes were used by 15% and 5% of produced mAbs, respectively (Fig. 2; Table S1, S2 and S3). The pattern of Ig gene usage by non-HIV-1 mAbs was very similar to Abs produced from single B cells derived from four healthy individuals as reported by a separate published study [21] (Fig. 2).

There is partial statistical evidence that the two groups of mAbs might differ specifically in their usage of VH3 and VH1 genes. In the non-HIV-1 group of mAbs the usage of VH3 is dominant over the VH1 gene (p = 0.0003, N = 58) and similarly in control mAbs. In contrast, there is no such dominance in the anti-HIV-1 mAbs which preferentially use VH1 over VH3 genes (p = 0.50, N = 10). The VH1 genes in anti-HIV-1 are more frequently used than in non-HIV-1 mAbs as tested by binomial tests (p = 0.055) (Fig. 2).

The reduction of VH3 and/or dominance of VH1 family gene usage by anti-HIV-1 Env mAbs was observed previously [9], [10], [11], [12], [15], but the normal pattern of Ig genes used by non-HIV-1 Env mAbs derived from the same HIV-1 infected donor is a new observation.

## Discussion

Virus-like particles have previously been utilized to isolate HIV-specific B cells for analysis of production of mAbs [18]. Although the comparative utility of VLPs as B cell probes compared with rationally-designed epitope scaffolds [22], gp140-based reagents [10], or neutralization screens [23] has not been fully evaluated, the potential for VLPs to correctly present the native trimer is attractive for this purpose. VLPs may be particularly well-suited to identify reactivity against quaternary epitopes [24], [25]. The current report extends previous findings employing VLPs for B cell isolation from HIV-infected individuals, and establishes that neutralizing antibodies can be identified with this technique. The finding that reactivity against gp41 epitopes was selected by a VLP-based immunogen also raises the possibility that antibodies that recognize both the MPER and the lipid bilayer of the VLP, similar to the activity of 2F5 [26] might be detected by VLPs in future screening.

For this reason we have used the VLPs to study the antibody repertoire and Ig gene usage by anti-HIV-1 Env mAbs produced from selected single B cells using molecular methods. We have developed 10 anti-HIV-1 mAbs with neutralizing activity to CD4bs and V3 and non-neutralizing gp41 mAbs. It confirms that VLPs expose the Env regions, for example CD4bs and V3, which are immunogenic and can induce neutralizing Abs upon vaccination.

Production of anti-V3 mAbs came with some surprise as the V3 region is supposed to be cryptic on the JRFL virus according to a previously published study [27]. It indicates however that the V3 loop is expressed to some extent on the JRFL virus surface, which is adequate for binding both the B cells and antibodies, but the affinity of binding is not sufficient for neutralization, possibly due to steric hindrance. Of the two anti-V3 mAbs tested, only one, 3b38, neutralized the JRFL.JB pseudovirus but just barely below 50 µg/ml for IC50 (Table 3). A similar pattern was observed for the anti-CD4bs mAbs as none of three such mAbs, selected by JRFL-VLPs, neutralized the corresponding pseudovirus.

Sorting of the stained IgG^+^ B cells with JRFL-VLP resulted in the production of 34 mAbs in which 10 mAbs (29%) were specific to HIV-1 Env while the remaining 24 mAbs had undefined specificities, but not to HIV-1 Env antigens. The high percentage of non-HIV-1 mAbs could be related to various factors which determine the non-specific binding of VLPs to B cells but which were not tested in this study with the exception of polyreactivity. The percentage of polyreactive mAbs among those which were non-specific was quite low as only three out of 58 (5%) mAbs bound to native 293T cells (data not shown) while among anti-HIV-1 mAbs this percentage was higher, three of 10 (30%) bound to native 293T cells and four of 10 (40%) bound to cardiolipin (Table 1). Interestingly, three out of four gp41 mAbs bound to cardiolipin and, as shown in another study, the mAbs to gp41 cluster II epitopes located close to the cell membrane are particularly prone to polyreactivity [28]. The frequency of polyreactivity in our anti-HIV-1 mAbs is comparable but not the same as by another study where 75% of 134 human anti-HIV-gp140 mAbs cloned from single B cells derived from six patients bound to various autoantigens, including cardiolipin [29].

Immunoglobulin gene usage analysis usually requires a large panel of antibodies to determine any predominance, but even in the small group of 10 anti-HIV-1 Env mAbs developed in this study, we have noticed preferential usage of VH1 over VH3 family genes which confirmed the biased usage observed in several other studies. For instance, the VH1 gene family was exclusively used by human mAbs against CD4i and preferentially by anti-gp41 mAbs, as well as by other anti-HIV-1 mAbs [9], [10], [15]. The reduced usage of VH3 family genes was observed among human mAbs against CD4bs, CD4i, gp41 and V3 developed by cellular methods (based on EBV transformation and fusion of reactive B cells with myeloma cells), while the VH5-51 gene was dominant among anti-V3 mAbs [9], [10], [12], [13], [14]. It is noted that the biased usage of Ig genes is observed in mAbs directed to the neutralizing face of gp120 as such mAbs were preferentially selected by the screening methods using V3 fusion proteins, trimerized gp140 and VLPs. It is possible that the non-neutralizing mAbs may have different Ig gene usage but they were not yet analyzed.

However, in 58 non-HIV-1 mAbs developed using the Gag-VLPs and an additional two other VLPs, the usage of Ig genes was not biased but, rather, identical to the pattern observed in Abs derived from single B cells of healthy subjects. As both panels of mAbs, anti-HIV-1 and non-HIV-1 specific, were produced from single B cells derived from one infected individual, this suggests that biased usage of Ig genes by HIV-1 antibodies is related to antigen selection of naïve B cells with receptors encoded by particular VH1 family genes which provided a more optimal fit. Apparently the VH3 family genes are not preferentially used by anti-HIV-1 Abs compared to non-HIV-1 Abs.

The reduced usage of VH3 family genes by anti-HIV-1 Abs was thought to be the result of the depletion of B cells with Ig receptors encoded by VH3 genes, which are a natural ligand for HIV gp120 [16]. It was found that gp120 as a superantigen has the ability to bind to a conserved surface on the VH3 region of Ig molecule mAbs [10], [12], [13]. It was hypothesized that the VH3 B cells bearing HIV gp120 can become a target for cytotoxic T cells which would eliminate these B cells, as being HIV-1 infected. If this is what occurs, then we would observe that B cells producing non-HIV-1 Abs encoded by the VH3 family genes will be also eliminated. Our study showed, however, that this was not the case in this HIV-1 infected donor, as the non-HIV-1 Abs were preferentially using VH3 genes, indicating that the corresponding B cells were not eliminated. It is possible that gp120 shed from virions can bind as superantigens to VH3 B cells but, most likely, the amount of bound gp120 is not sufficient for the B cells depletion mediated by effector T cells. Eventually this may occur in patients in the advanced stages of disease because, in one of the first papers which reported this phenomenon, the B cells expressing the VH3 gene segment were more reduced in patients with AIDS symptoms than in asymptomatic HIV-1 infection [10], [12], [13], [14].

Our observation is supported by an experimental study in rhesus macaques infected with chimeric viruses expressing HIV-1 Env proteins on a simian immunodeficiency virus (SIVmac) backbone (SHIV) [30]. During the primary infection with SHIV, the average representation of VH3 bearing B lymphocytes did not change suggesting that gp120 did not deplete the VH3 repertoire of B cells [30]. Furthermore, we analyzed the data from the study by Scheid et al [10], [12], [13] who developed gp140 non-binding mAbs along with anti-HIV-1 Env Abs and 45 of 92 (49%) of these non-HIV-1 Abs preferentially used VH3 family genes, supporting our similar observation.

We thus postulated that biased Ig gene usage by anti-HIV-1 mAbs is related to preferential selection of some VH-encoded Abs by the particular antigen and is not the result of compensation to depletion of VH3 B cells, as the gp120 superantigen hypothesis would suggest.

## Materials and Methods

### Ethics Statement

The study was approved by the New York University School of Medicine Institutional Review Board. A volunteer signed written approved informed consent forms prior to participating in the study.

#### Study participant

The HIV-1 infected individual had been seropositive and asymptomatic for at least 15 years and had CD4 T-cell counts of 378 per µl and viral load 22,000 RNA copies/ml. Patient was presumably infected with clade B virus based on the residences in the New York City area.

### Human anti-HIV-1 and Control Monoclonal Antibodies

Sixty-eight human recombinant mAbs, including 10 anti-HIV-1 and 58 non-HIV-1 envelope (Env) mAbs, were produced from single B cells derived from a volunteer. The mAbs were produced using the molecular techniques (see below) according to the described method [19]. In addition, human mAbs: 447-52D (anti-V3) [31], 654 (anti-CD4bs) [32], 167-7 (anti-gp41) [20] and 4.8D (anti-CD4i) [33] were used as positive controls while human mAb 1418 (anti-parvovirus B19) [34] served as a negative control.

### Recombinant Proteins

Recombinant gp120_MN_ and gp41_MN_ were purchased from Immunodiagnostics, Inc. (Woburn, MA). The recombinant gp120_HXB2_ mutant was kindly provided by Dr. Catarina Hioe, NYU School of Medicine. It contains two site mutations, D368R which abrogate binding of anti-CD4bs mAbs [35] and N448Q which eliminates the glycosylation site flanking the CD4 T cell epitope cluster in the C4 region [36].

### Green-fluorescent Protein-labeled Virus-like Particles (GFP-VLPs)

GFP-tagged VLPs were used for staining and single-cell sorting of Env-specific and non-specific B cells. The engineered GFP-tagged Vpr VLPs containing the Env proteins from JRFL or BaL and also one without Env were produced by 293 cells that have been stably transfected with multiple inducible promoter-driven constructs: Gag, Vpr-GFP, and JRFL or BaL *env* genes. The clonal cell line was selected based on production of VLPs with completely processed gp120/gp41 (cleaved gp160) as recently described [18]. Western blot analysis and blue native gel electrophoresis assays revealed the presence of cleaved gp120 and native trimers on the BaL-VLPs and JRFL-VLPs, respectively [18]. Induction with doxycycline allows efficient production of VLPs, which are purified using either iodixanol gradients or sedimenting through sucrose gradients as described [25].

### Sorting VLP-reactive B Cells

VLPs were used for selection of single IgG^+^ memory B cells specific to HIV-1 Env proteins as well as non-specific negative controls. PBMCs were isolated from the heparinized blood of an asymptomatic HIV-infected individual by Ficoll Hypaque density gradient centrifugation. The B cells were enriched using anti-CD19 magnetic beads (Milteyni Biotec, Auburn, CA) and then were stained on ice for 30 minutes with GFP-VLPs expressing either JRFL or BaL Env proteins. The enriched B cells contain usually >90% of B cells according to manufacturer’s protocol. The Gag-GFP-VLP (without Env) was used as a control for non-specific binding of VLPs to B cells. The VLPs with or without Env proteins were normalized by p24 and the VLPs with same concentration of p24 were used for staining the enriched B cells. After washing with PBS +1%BSA, the VLP stained cells were incubated again on ice for 30 minutes with anti-human IgG (Fc)-Phycoerythrin (PE) and a memory B cell marker anti-CD27-Allophycocyanin (APC). During single cell sorting, the first gate was set to select for viable B cells and the next gate was set to select for memory B cells (APC-CD27^+^). Then gating was done on the CD27^+^ memory B cells to select for those that were IgG^+^ and also bound to VLPs. Single cell sorting of a CD27^+^/IgG^+^/GFP^+^ population was performed using the FACSAria (BD Biosciences, San Jose, CA) in the CFAR Flow Cytometry Core at Bellevue Hospital (part of NYULSoM). The single IgG memory B cells that bound to VLPs were sorted into 96-well PCR plates (Thermo Scientific, Pittsburgh, PA) containing reverse transcriptase buffer, DTT, and RNAsin (Promega Corporation, Madison, WI), frozen on dry ice and stored at −80°C.

### RT-PCR Amplification of VH and VL Regions from Single B Cells

This method was performed according to the technique described by Tiller et al. [19]. Briefly, total RNA from single cells was reverse transcribed in the original 96-well sorting plate in nuclease-free water (Qiagen Inc, Valencia, CA) using a random hexamer primer (Invitrogen, Carlsbad, CA), dNTP mix (Invitrogen), DTT (Invitrogen), Igepal CA-630 (Sigma-Aldrich, St. Louis, MO), RNAsin (Promega), Prime RNAse Inhibitor (Eppendorf) and Superscript III reverse transcriptase (Invitrogen). IgH, Igκ and Igλ variable gene transcripts were amplified independently by nested PCR.

All PCR reactions were performed in 96-well plates containing the external primers mix for first round PCR or internal primers mix for second round PCR, dNTP mix (Invitrogen), and HotStar Taq DNA polymerase (Qiagen). The primers used in these assays were described [19]. Aliquots of the VH, Vκ and Vλ chain from the second PCR products were purified with ExoSAP-IT (USB, Santa Clara, CA) and sequenced with the reverse primer. The sequence data were analyzed using Pregap4 and BioEdit softwaresalong with the International ImMunoGene Tics (IMGT) information system (http://imgt.cines.fr).

### Cloning VH and VL into Expression Vectors

The human Igγ1, Igκ or Igλ expression vectors were kindly provided by Dr. Michel Nussenzweig (Rockefeller University, New York, NY). Ligations were performed with T4 DNA-Ligase (Invitrogen), PCR product and a linearized vector. Competent *E.coli* DH10B bacteria (Invitrogen) were transformed at 42°C with 5 µl of the ligation product. Colonies were screened by PCR. Plasmid DNA was isolated from bacterial cultures grown in Terrific Broth (Invitrogen) containing ampicillin using QIAprep Spin columns (Qiagen).

### Cotransfection of Ig Vectors into 293T Cells for Production of Recombinant mAbs

The 293T human embryonic kidney cells (ATCC) were transfected using FuGENE HD (Roche) with equal amounts of 0.5 µg/ml of IgH and corresponding IgL chain expression plasmids. The cells were then cultured for 3 days in DMEM supplemented with 10% fetal bovine serum. The culture supernatants were harvested and IgG Abs were purified by Protein A chromatography using Hi-Trap IgG columns (GE Healthcare). The IgG concentrations were determined by quantitative ELISA [37].

### TZM-bl Neutralization Assay

Six recombinant mAbs specific to the V3 region and CD4 binding site (CD4bs) were tested for neutralizing activities against 41 pseudoviruses using the TZM-bl cell line as described [38], [39]. Briefly, 2-fold serial dilutions of mAbs, starting from 50 µg/ml, were preincubated with the virion-containing culture supernatants and incubated 48 hrs with TZM-bl cells expressing CD4, CXCR4 and CCR5. The virus infectivity was determined by measuring the luciferase activity in the cell lysates. The reduction of infectivity was expressed as percent neutralization by comparing the enzyme activity, as relative light units, in the presence of mAbs versus absence of mAbs [39], [40].

### Binding Assays

A standard ELISA was used to determine binding of mAbs to gp120_MN_ and gp41_MN_ as described [41]. Briefly, ELISA plates were coated overnight at 4°C with antigen, blocked with 2% BSA in PBS, and then incubated for 1.5 h at 37°C with human mAbs at 10 µg/ml; the bound mAbs were detected by incubation with alkaline phosphatase (AP)-conjugated goat anti-human IgG (Fc) followed by incubation with substrate and the plates were read at 405 nm.

IgG quantitation was also performed by ELISA as described [37]. Briefly, ELISA plates were coated with goat anti-human IgG (Fc) and incubated with culture supernatants. Bound IgG was detected with alkaline phosphatase-conjugated goat anti-human IgG (Fc). Affinity-purified human IgG (Sigma) was used to produce standard curves.

Binding of mAbs to cardiolipin was tested by ELISA. Briefly, 60 µl cardiolipin at a concentration of 45 µg/ml in ethanol was coated onto ELISA plates by evaporation at 4°C overnight. Plates were then blocked to prevent non-specific binding of immunoglobulins with 100 ul 2% BSA for 2 hrs at room temperature then the procedure was followed according to the standard ELISA as described above.

The effect of soluble CD4 (sCD4) on mAbs binding to gp120 was tested by standard ELISA; sCD4 at concentration of 2 µg/ml was incubated with gp120 prior to mAbs binding to gp120. Binding of human mAbs was detected using alkaline phosphates conjugated goat anti-human IgG (Fc).

### Flow Cytometric Analysis

Binding of mAbs to Env-transfected cells. 293T cells were transfected either with the JRFL or SF162 *env* expression vector and pSV-*rev* vector using FuGENE HD. The cells were harvested 36 hrs post-transfection and incubated with 10 µg/ml of human mAb for 30 minutes at 4°C followed by staining with goat F(ab)2 anti-human IgG (γ) conjugated with PE (Caltag Laboratories, Burlingame, CA). The cells were then fixed with 2% paraformaldehyde and analyzed by flow cytometry using a FACSCalibur (Becton Dickinson, San Jose, CA) as described [42].

### Statistical Analysis

Binomial and Chi-Squared tests were used to compare the Ig gene usage by human mAbs.

## Supporting Information

Figure S1 **Binding of mAbs selected by JRF-VLPs and control anti-HIV-1 mAbs to gp120_MN_ alone and preincubated with soluble CD4.** The study was performed by standard ELISA using 96-well plates coated with gp120_MN_ at a concentration of 1 µg/ml and incubated with sCD4 at a concentration of 2 µg/ml prior to incubation with mAbs at 10 µg/ml. Binding of human mAbs was detected using alkaline phosphates conjugated goat anti-human IgG (Fc).
(DOCX)

Table S1 **Human non-HIV-1 mAbs produced from single IgG^+^ B cells selected using JRFL-VLPs.** This table depicts a list of 24 human mAbs produced from single B cells derived from an HIV-1 infected individual (the same as in Table S2 and S3) and stained with JRFL-VLPs but which did not show any binding activity to HIV-1 Env proteins. Each mAb is unique as determined by the usage of IGHV and IGLV genes, length and sequence of the CDR H3 domain.
(DOCX)

Table S2 **Human non-HIV-1 mAbs produced from single IgG^+^ B cells selected using BaL-VLPs.** The listed 19 mAbs were produced from B cells selected with VLPs expressing HIV-1_BaL_ Env proteins and did not show any binding activity to Env proteins.
(DOCX)

Table S3 **Human non-HIV-1 mAbs selected from single IgG^+^ B cells using Gag-VLPs.** This table shows a list of 15 mAbs, selected by VLPs without HIV-1 Env proteins, which did not react with Env proteins.
(DOCX)
