# Supplementary material for: Different Pattern of Immunoglobulin Gene Usage by HIV-1 Compared to Non-HIV-1 Antibodies Derived from the Same Infected Subject
Source: PLoS One. 2012 Jun 25;7(6):e39534. doi: 10.1371/journal.pone.0039534 (PMC3382572; doi:10.1371/journal.pone.0039534)
Supplement: Figure S1 — Binding of mAbs selected by JRF-VLPs and control anti-HIV-1 mAbs to gp120MN alone and preincubated with soluble CD4. The study was performed by standard ELISA using 96-well plates coated with gp120MN at a concentration of 1 µg/ml and incubated with sCD4 at a concentration of 2 µg/ml prior to incubation with mAbs at 10 µg/ml. Binding of human mAbs was detected using alkaline phosphates conjugated goat anti-human IgG (Fc). (DOCX) [file pone.0039534.s001.docx]

**Supporting Information**

Figure S1.
