# Supplementary material for: Different Pattern of Immunoglobulin Gene Usage by HIV-1 Compared to Non-HIV-1 Antibodies Derived from the Same Infected Subject
Source: PLoS One. 2012 Jun 25;7(6):e39534. doi: 10.1371/journal.pone.0039534 (PMC3382572; doi:10.1371/journal.pone.0039534)
Supplement: Table S1 — Human non-HIV-1 mAbs produced from single IgG+ B cells selected using JRFL-VLPs. This table depicts a list of 24 human mAbs produced from single B cells derived from an HIV-1 infected individual (the same as in Table S2 and S3) and stained with JRFL-VLPs but which did not show any binding activity to HIV-1 Env proteins. Each mAb is unique as determined by the usage of IGHV and IGLV genes, length and sequence of the CDR H3 domain. (DOCX) [file pone.0039534.s002.docx]

Table S1. Human non-HIV-1 mAbs produced from single IgG^+^ memory B cells selected using JRFL-VLPs.

| # | mAb | IGHV | CDR H3 | IGLV | CDR L3 |
| --- | --- | --- | --- | --- | --- |
| 1 | 3c75 | 1-2 | ARAGGDYSKYEYFFDY | [L1-44](file://C:\Users\Mirek\AppData\Local\Microsoft\Windows\Miroslaw%20K.%20Gorny\Local%20Settings\Documents%20and%20Settings\Miroslaw%20K.%20Gorny\Local%20Settings\Temporary%20Internet%20Files\Local%20Settings\Temporary%20Internet%20Files\OLK1F\mi-tables-paper\attachments_2010_09_13\gene%20usage.xlsx#RANGE!Vcomment) | ATWDDSLEQG |
| 2 | 3c66 | 1-3 | AREGEFMVYFDY | K4-1 | QQYYNKPYT |
| 3 | 3c13 | 1-18 | GRNGGFGELFPFDY | K1-8 | QQYYSYPYT |
| 4 | 3c11 | 1-18 | ARLGPHNWFDL | K4-1 | QQYYSTQLT |
| 5 | 3c58 | 1-69 | ARGHGRLVGATRFDP | L2-23 | SSYVGNSTHV |
| 6 | 3b1 | 3-7 | VREVQEPSQRFDY | K1-39 | QQSHSTPRT |
| 7 | 3b80 | 3-11 | ARVLWYYESSGNAFDI | K3-11 | QQRSNLYT |
| 8 | 3b65 | 3-23 | AKDLNGSGSPSGLDAFEI | L3-1 | QGWDNSTSYV |
| 9 | 3b94 | 3-23 | AKAGRFCGGDCYFAF | [K3-20](file://C:\Users\Mirek\AppData\Local\Microsoft\Windows\Miroslaw%20K.%20Gorny\Local%20Settings\Documents%20and%20Settings\Miroslaw%20K.%20Gorny\Local%20Settings\Temporary%20Internet%20Files\Local%20Settings\Temporary%20Internet%20Files\OLK1F\mi-tables-paper\attachments_2010_09_13\gene%20usage.xlsx#RANGE!Vcomment) | QQYGSSPLT |
| 10 | 3c32 | 3-23 | AKRAQQWLDPPPYWFDP | K4-1 | QQYYSTPLT |
| 11 | 3c45 | 3-23 | AKDIHSWMYSSLEGDYYYGMDV | L3-1 | QAWDSSTAYV |
| 12 | 3b70 | 3-23 | AKVDCSGGTCDSGVTT | L3-25 | QSADSSGTFV |
| 13 | 3b36 | 3-30 | RDPPRAATPYFDFW | K2D-29 | MHSIQFPPITF |
| 14 | 3c85 | 3-30 | GRGKSGYYYDSSGYYWDYFDY | L2-11 | SYGGTYSRKV |
| 15 | 3b53 | 3-30 | ARDLVGATPRY | K4-11 | QQYYRSPPT |
| 16 | 3c62 | 3-30 | ARDPGYIGAAAGYFGN | L1-47 | VTWDDSLIGHV |
| 17 | 3c63 | 3-48 | ARGRGYSSAWSLDY | K1-39 | QQTYLAPWT |
| 18 | 3b7 | 4-30 | ARADGDDVVFDL | K3-20 | QQYISSPWT |
| 19 | 3c71 | 4-30 | AREYCSGGSCLFDY | L3-21 | QGWDNSIDHSYV |
| 20 | 3b16 | 4-34 | ARVRRDDFWSSYYGGFDRYFDY | K3-15 | QQYNNWPPLT |
| 21 | 3c18 | 4-34 | ARGAARPDY | K1-39 | QQSYRTPRT |
| 22 | 3c65 | 4-34 | VRGAGGSSGYLLLDF | [K1-6](file://C:\Users\Mirek\AppData\Local\Microsoft\Windows\Miroslaw%20K.%20Gorny\Local%20Settings\Documents%20and%20Settings\Miroslaw%20K.%20Gorny\Local%20Settings\Temporary%20Internet%20Files\Local%20Settings\Temporary%20Internet%20Files\OLK1F\mi-tables-paper\attachments_2010_09_13\gene%20usage.xlsx#RANGE!Vcomment) | LQDYTYPKT |
| 23 | 3c27 | 4-34 | ARGLSQGYTYAPPFDS | [K1-5](file://C:\Users\Mirek\AppData\Local\Microsoft\Windows\Miroslaw%20K.%20Gorny\Local%20Settings\Documents%20and%20Settings\Miroslaw%20K.%20Gorny\Local%20Settings\Temporary%20Internet%20Files\Local%20Settings\Temporary%20Internet%20Files\OLK1F\mi-tables-paper\attachments_2010_09_13\gene%20usage.xlsx#RANGE!Vcomment) | QQNNVYSPT |
| 24 | 3c39 | 5-51 | ARSPVGATRRCDAFDI | L4-69 | QTWGTGVHVV |
