# Supplementary material for: Different Pattern of Immunoglobulin Gene Usage by HIV-1 Compared to Non-HIV-1 Antibodies Derived from the Same Infected Subject
Source: PLoS One. 2012 Jun 25;7(6):e39534. doi: 10.1371/journal.pone.0039534 (PMC3382572; doi:10.1371/journal.pone.0039534)
Supplement: Table S2 — Human non-HIV-1 mAbs produced from single IgG+ B cells selected using BaL-VLPs. The listed 19 mAbs were produced from B cells selected with VLPs expressing HIV-1BaL Env proteins and did not show any binding activity to Env proteins. (DOCX) [file pone.0039534.s003.docx]

Table S2. Human non-HIV-1 mAbs produced from single B cells selected using

BaL-VLPs

| # | mAb | IGHV | CDR H3 | IGLV | CDR L3 |
| --- | --- | --- | --- | --- | --- |
| 1 | 4a84 | 3-7 | AKGPAIVIIPTXMNFYSYGMDV | K1-39 | QQSHSTPFLS |
| 2 | 4a2 | 3-13 | ARDRGSCSSTSCYVGHFDL | K3-11 | QQRSNWPPLT |
| 3 | 4a18 | 3-15 | TTGGGRHYYFDY | L2-14 | SSYTTSSTRV |
| 4 | 4a38 | 3-23 | AKLHYSDYYYDSSGYLSPWYFDL | K1-33 | QQYDNLPIT |
| 5 | 4a39 | 3-23 | AKDLTSGFIFSYNYYYGMDV | L2-14 | GSYAPNIPVV |
| 6 | 4a27^a^ | 3-30 | VKDERRRYCSSNICYNVYHGMDV | K2-28 | MQALQTPRT |
| 7 | 4a91^a^ | 3-30 | AKDERRKYCSSNTRCYNVYHGIDV | K2-28 | MQALQTPRT |
| 8 | 4a86 | 3-30 | AKTHTWFGELLAFDY | K1-5 | QQYNSYSYT |
| 9 | 4a93 | 3-33 | ARDRSSSSVNWFDP | K1-39 | QQSYSTLYT |
| 10 | 4a28 | 3-48 | VSGYTYGYYFDY | K3-20 | QQYGSSPKYT |
| 11 | 4a92 | 3-48 | AKTGNWNDGYFDY | K1-39 | QQSYSTLYT |
| 12 | 4a3 | 3-49 | TRDYFDSSSLGTPVY | L3-25 | QSADSRGSYVV |
| 13 | 4a65 | 4-31 | ARGLNYYGSGFFDH | K3-20 | QQYGSSPTT |
| 14 | 4a81 | 4-34 | ARRSGYSFDY | K1-39 | QQSYISPYT |
| 15 | 4a50 | 4-39 | ARLYSSMYDILTGFYYFDY | K4-1 | QQYYSTPLT |
| 16 | 4a33 | 4-59 | ARQPEYGGPWYFDL | L2-18 | SSYRSNSTLIHVV |
| 17 | 4a17 | 4-61 | AGLEITYNSDWSPFDY | K3-20 | QQYGGSPPT |
| 18 | 4a68 | 4-61 | ARDHREWFGELLYSRGMDV | K1-16 | QQYNTYPPT |
| 19 | 4a4 | 5-51 | ARTSYYYDSSPYHPGLFDY | K4-1 | CQQYYSTPWTF |

^a^These two mAbs may have originated from one B cell clone
