# Supplementary material for: Different Pattern of Immunoglobulin Gene Usage by HIV-1 Compared to Non-HIV-1 Antibodies Derived from the Same Infected Subject
Source: PLoS One. 2012 Jun 25;7(6):e39534. doi: 10.1371/journal.pone.0039534 (PMC3382572; doi:10.1371/journal.pone.0039534)
Supplement: Table S3 — Human non-HIV-1 mAbs selected from single IgG+ B cells using Gag-VLPs. This table shows a list of 15 mAbs, selected by VLPs without HIV-1 Env proteins, which did not react with Env proteins. (DOCX) [file pone.0039534.s004.docx]

Table S3. Human non-HIV-1 mAbs selected from single B cells selected using Gag-VLPs.

| # | mAb | IGHV | CDR H3 | IGLV | CDR L3 |
| --- | --- | --- | --- | --- | --- |
| 1 | 3d48 | 1-2 | AKEYDVLSYAMDV | [L2-14](file://C:\Users\Mirek\AppData\Local\Microsoft\Windows\Miroslaw%20K.%20Gorny\Local%20Settings\Documents%20and%20Settings\Miroslaw%20K.%20Gorny\Local%20Settings\Temporary%20Internet%20Files\Local%20Settings\Temporary%20Internet%20Files\OLK1F\mi-tables-paper\attachments_2010_09_13\gene%20usage.xlsx#RANGE!Vcomment) | RSFTTRGIIV |
| 2 | 3d30 | 1-6 | ARVLTSIGPNGMDV | [K3-20](file://C:\Users\Mirek\AppData\Local\Microsoft\Windows\Miroslaw%20K.%20Gorny\Local%20Settings\Documents%20and%20Settings\Miroslaw%20K.%20Gorny\Local%20Settings\Temporary%20Internet%20Files\Local%20Settings\Temporary%20Internet%20Files\OLK1F\mi-tables-paper\attachments_2010_09_13\gene%20usage.xlsx#RANGE!Vcomment) | QQYVGPPWT |
| 3 | 3d39 | 1-18 | ARDPAPRLKWNHYFDY | K1-5 | QQYNDYST |
| 4 | 3d50 | 1-69 | ARAKAPPLYTTSWYEIDF | K3-20 | QHYASSPIT |
| 5 | 3d89 | 3-15 | TTAHYYSDETYFDF | K4-1 | HQYYTVSFS |
| 6 | 3d22 | 3-15 | RVPHLVWLGETLHYYGVD | L1-51 | GTWDSSLSAYV |
| 7 | 3d38 | 3-15 | TTADLMVRGGVPDF | K3-15 | QQYNKWWT |
| 8 | 3d18 | 3-20 | ARGATPVTNYFDS | K3-15 | QQYNNWPRT |
| 9 | 3d44 | 3-30 | ARDGTYGEGSWRDYYYYGMDV | K1-12 | QQANSFPLT |
| 10 | 3d73 | 3-49 | TREQSLNKYSSSWYADY | [K4-1](file://C:\Users\Mirek\AppData\Local\Microsoft\Windows\Miroslaw%20K.%20Gorny\Local%20Settings\Documents%20and%20Settings\Miroslaw%20K.%20Gorny\Local%20Settings\Temporary%20Internet%20Files\Local%20Settings\Temporary%20Internet%20Files\OLK1F\mi-tables-paper\attachments_2010_09_13\gene%20usage.xlsx#RANGE!Vcomment) | QQYYTTPRT |
| 11 | 3d11 | 3-66 | AEEGIAVAGRGY | K1-39 | QQSYSTPLT |
| 12 | 3d34 | 4-31 | ASHSTPYAAAGYFDY | K1-12 | QQANSFLLFT |
| 13 | 3d94 | 4-59 | ARARKAGNANTWPRQLDP | K2-28 | MQSLQIPS |
| 14 | 3d42 | 4-61 | ARERGTVYVSAGGFDI | K3-11 | QQRTNWLLT |
| 15 | 3d60 | 5-a | ARNRLWNPEDY | K1-27 | QKYNSAPLT |
